# An action research partnership in an urban Texas county to explore barriers and opportunities for collaborative community health needs assessments

**DOI:** 10.3389/fpubh.2023.1244143

**Published:** 2023-10-11

**Authors:** Marcela Nava, Amanda S. English, Linda Fulmer, Katherine Sanchez

**Affiliations:** ^1^School of Social Work, The University of Texas at Arlington, Arlington, TX, United States; ^2^Institute for Implementation Science, The University of Texas Health Science Center at Houston, Houston, TX, United States; ^3^Healthy Tarrant County Collaboration, Fort Worth, TX, United States; ^4^Center for Applied Health Research, Baylor Scott and White Research Institute, Dallas, TX, United States

**Keywords:** community health needs assessment, nonprofit hospitals, decision-making, political conservatism, participatory action research, community benefit

## Abstract

**Background:**

The Affordable Care Act mandated triennial community health needs assessments (CHNAs) for greater nonprofit hospital accountability in responding to community health needs. Over 10 years later, hospital spending on community benefits remains largely unchanged. While greater collaboration in CHNA implementation can increase hospital investment in community-based initiatives, nonprofit hospitals in conservative states are subject to policy, political, and economic factors that inhibit public health partnerships and magnify existing disparities in health care access. This participatory action research study explores the decision-making environment of collaborative CHNA implementation within a group of nonprofit hospitals in a north Texas urban county.

**Methods:**

In 2017 faculty from an urban anchor institution initiated an academic-community partnership with a coalition of nonprofit hospitals, public health departments, and academic institutions. An interdisciplinary research team engaged in multi-method document review and qualitative data collection to describe historical barriers for local CHNA processes and develop practical strategies for joint CHNA initiatives. Local CHNA documents were first reviewed through team-based content analysis and results applied to develop a qualitative study protocol. Key informants were recruited from county-based nonprofit hospitals, community-based nonprofit organizations, and public health systems. Seventeen senior- and mid-level professionals participated in semi-structured research interviews to describe their perspectives relating to CHNA-related planning and implementation decisions. Through iterative data collection and analysis, the research team explored CHNA-related knowledge, experiences, and processes. A constructivist lens was subsequently applied to examine historical barriers and future opportunities for local collaboration.

**Results:**

Findings reveal CHNA implementation is a multi-stage cyclical process in organizational environments with accountability to a wide range of public and private stakeholders. This promotes varied levels of inclusivity and conservatism in data collection and community benefit implementation. Decisions to collaborate are hindered by competing priorities, including compliance with existing guidelines, administrative simplicity, alignment with health care service delivery, and efficient resource use. Efforts to promote greater CHNA collaboration may be facilitated through intentional alignment with organizational priorities and clearly communicated benefits of participation for leaders in both public and private nonprofit health systems.

**Discussion:**

We consider implications for policymakers and health systems in restrictive political environments and advance a conceptual framework for greater CHNA collaboration.

## 1. Introduction

Nonprofit hospitals in the US must provide services and activities that constitute “community benefit” in order to maintain their federal tax-exempt status (1). While overall spending on community benefit is greater than what would be paid in federal taxes, historically over 85% of this spending has subsidized the costs of charitable health care services rather than community health activities (2). In response to widespread demand for greater hospital accountability to respond to community needs (3, 4), the Affordable Care Act (ACA) created new regulations for nonprofit hospitals to conduct triennial community health needs assessments (CHNAs) and intervention plans (5). Despite widespread compliance with this mandate, spending did not increase as a percentage of hospital expenses following the ACA (6) and recent studies find few differences between nonprofit and for-profit hospitals in charitable spending patterns (7).

CHNA implementation consists of various processes with the potential to reduce, maintain, or exacerbate systematic barriers to community health and contribute to health disparities (8, 9). Under present guidelines, hospitals have significant discretion in how to define target communities, collect and engage in data analysis, prioritize community health needs, and select community benefit activities. At the same time, hospital leaders had limited experience with community health assessments prior to the ACA (10), and some report up to tens of thousands of hours of personnel time to comply with existing guidelines (11). Greater collaboration can promote greater efficiencies (12), increase hospital spending on community benefit activities (13), encourage evidence-based decision-making to address community health outcomes (14), and promote a shared understanding of community health (15). However, content analyses of CHNA and implementation plan reports reveal limited collaboration with other hospitals or public health agencies (16, 17). This can generate duplicative data collection (18), reduce transparency (19), and foster misalignment with other community health activities (20).

While there is evidence that federal regulations have increased nonprofit hospital partnerships with external organizations (21), hospitals in the US south report fewer collaborations with public health, governmental, and non-profit organizations (22). Seven out of ten states that remain opposed to Medicaid expansion are located in the south (23), where Republican-controlled legislatures cite concerns of long-term government dependence and infringement on states’ rights (24). Nonprofit hospitals with limited access to the benefits of Medicaid expansion were less likely to experience a reduction in unpaid bills following the ACA (25, 26). They are more likely to subsidize uncompensated care than invest in community health initiatives (27, 28) and report less overall community benefit spending (29). Scholars have pointed out the shortcomings of current federal guidelines to ensure meaningful community partnerships for nonprofit hospital accountability (18, 26, 30–32), and certain states have extended guidelines for community involvement beyond the federal mandate (33–35). However, it is essential to consider whether greater CHNA regulations may generate superficial compliance in politically conservative regions (36, 37). Given increasing partisan polarization, conservative state policymakers may limit enforcement of policies that challenge ideological norms of limited government and personal responsibility for health (24, 38, 39). Facing regulatory pressure to invest in more widespread and collaborative needs assessments, hospital administrators may choose to raise income ceilings or reduce overall charitable care spending to cover additional costs (34, 40). As a result, this may lower healthcare quality for high-cost uninsured patients (41–44) and amplify mistrust of health systems in marginalized communities (44).

Many states participate in the 1115 Medicaid waiver program, which was authorized by the Social Security Act to encourage innovative approaches for extending healthcare services in regions with high uninsurance rates (45, 46). 1115 waiver programs provide states with considerable flexibility to pursue fiscally conservative policies, such as imposing restrictions on Medicaid recipients and encouraging the purchase of private plans through managed care organizations (47). Texas is home to the highest proportion of state Medicaid beneficiaries enrolled in managed care contracts (15). Despite active participation in the 1115 Medicaid waiver program (48), Texas is home to the largest number and percentage of uninsured residents in the United States (49). Outside of state-federal funding partnerships, some states have established minimum standards for community benefit spending (33). Texas is considered to have one of the most stringent community benefit spending policies in the country (4), requiring nonprofit hospitals to invest at least 5 % of net patient revenue (50). These guidelines, however, may disincentivize hospital spending above the threshold of compliance, particularly for hospitals in urban regions that sustain a higher demand for subsidized health care services (13, 51).

To advance meaningful community partnerships for CHNA implementation, it is essential to explore environmental conditions of nonprofit hospitals in regions with limited political support for public investment in health equity initiatives (52, 53). This requires methodological approaches that move beyond national and state-wide foci (54, 55) to engage local community stakeholders in the research process (53, 56). This multi-method participatory action research (PAR) study seeks to advance strategies for collaborative CHNA implementation in Tarrant County, the second-largest county in the Dallas/Fort Worth metroplex with a long-established reputation as “the largest reliably Republican county” in Texas (57). A team of academic partners and community health collaboration leaders engaged in a research-practice-policy partnership over a seven-year timeframe to accomplish three primary aims:

1. Conduct a document review of CHNAs from county-based nonprofit hospitals and community planning agencies.
2. Qualitatively assess challenges and opportunities for collaborative implementation.
3. Promote sensible CHNA collaboration efforts through a contextual understanding of the local social and political environment.

PAR is a pragmatic set of methodologies for collaboratively exploring social issues while simultaneously advancing practical solutions (58, 59). This is carried out through a cyclical and collaborative process that seeks to balance data-driven analysis with social action. PAR is inherently participative given its action-focused lens, which extends the role of community members as equitable partners throughout the research process. This ensures that studies are grounded in the needs and issues of communities and community-based organizations while facilitating collaborative learning to actively translate research findings into practice (60). To guide our study, we applied principles and guidelines adapted by the board of the Detroit Community-Academic Urban Research Center (61), a well-established hub of community-based participatory research (CBPR) projects that aim to build policy advocacy skills and advance policies through collaborative partnerships. These partnerships empower community stakeholders with knowledge and skills through circular co-learning processes of assessing community strengths and dynamics, identifying priority issues, design and conducting research studies, and interpreting and disseminating findings (62).

## 2. Methods

### 2.1. History of partnership

While federal CHNA guidelines provide hospitals with wide latitude in defining target communities (4), Texas guidelines for state tax exemptions explicitly require inclusion of the county in which the hospital is located (63). This obligation led to the creation of the Healthy Tarrant County Collaboration (HTCC) in 1997 as a hospital partnership to facilitate a county-specific needs assessment. HTCC persisted over the years with a mission to advance community health in Tarrant County. Drawing on the Spectrum of Prevention framework to identify and develop multifaceted initiatives that address gaps in fostering community health coalitions and networks (64), this organization operates as a tax-exempt organization governed by representatives of nonprofit hospitals, public health agencies, and universities. In 2016, two social work researchers from an academic anchor institution initiated an action research partnership with HTCC to assess local barriers and opportunities for collaborative CHNA implementation. This study was driven by HTCC’s 2016–2019 strategic goal to “increase the quality of primary data for Tarrant County for community health needs assessments and grant applications.” To establish an academic-community research partnership, we executed contracts to integrate HTCC’s Executive Director (Fulmer) and Steering Committee Chair (English) as part of the research team, leveraging critical expertise derived from their lengthy histories of organizational leadership in CHNA implementation and other community health initiatives. While these HTCC leaders did not engage in data collection to minimize perceived conflicts of interest, they actively participated in designing the research protocol and sampling methods, recruiting participants, analyzing data, developing dissemination materials, facilitating communication with HTCC governance and advisory groups, and advancing materials for publication.

### 2.2. CHNA document review

Beginning in early 2017, the research team conducted a web-based search to identify CHNAs that included Tarrant County in their target communities. We discovered a total of 22 CHNAs from organizational websites of five county-based nonprofit hospital facilities. For-profit hospitals were excluded as they are not bound to federal regulations that require community benefit spending and charitable care. Our search also revealed three additional CHNAs from the county public health department, a local United Way chapter engaged in community planning, and an 1115 waiver regional health partnership led by the local public hospital. Academic faculty and research students applied content analysis methods to review a total of 25 CHNAs completed in 2013–2014 and 2016–2017 (the first two rounds of CHNAs following the mandate). Documents were coded using Nvivo qualitative data software to identify specific elements or processes (e.g., electronic surveys, use of outside vendors) that characterized each report. These codes were reviewed by the research team and grouped into six descriptive categories: (1) knowledge and interpretation of key terms and mandates associated with the CHNA process; (2) roles, skillsets, and capacities of organizational leaders that engage in CHNA decision-making and implementation; (3) CHNA data collection planning and processes; (4) community health data interpretation and health issue prioritization; (5) community benefit strategy development; and (6) strengths and weaknesses of CHNA implementation processes. We then expanded each category to develop open-ended research questions for a semi-structured key informant interview protocol. The interview instrument elicited participant knowledge and perspectives through general questions (e.g., How does primary data influence implementation strategies?) as well as specific questions and probes based on leadership roles (e.g., How did you decide your…budget, timeline, vendors?) or operational roles (e.g., How was data collected through…surveys, focus groups, interviews?).

### 2.3. Participant recruitment

Recognizing that health care systems are not the only organizations conducting CHNAs, this study uses the term “organizations” to refer to both hospitals and key community partners, with specific references to health care systems where appropriate. We recruited decision-makers with oversight of CHNA planning or implementation in organizations identified through the document review. This included the local public hospital, four private nonprofit hospitals, a local United Way, and the county public health department. All the private nonprofit hospitals represented in our county-based sample belong to regional health systems with distinct geographic boundaries. Initial interviews included executive leaders with CHNA oversight, who identified additional participants with managerial or operational oversight. Beginning in January 2017, seventeen individuals participated in semi-structured research interviews over a six-month period. This included six individuals in leadership positions, eight individuals with data collection responsibilities, and one focus group interview with four individuals with data collection responsibilities. Participant characteristics, including education level, self-reported race and ethnicity, and gender, are provided in Table 1. This study was approved by The University of Texas at Arlington Institutional Review Board (2017–0255). Participants gave written informed consent and completed a personal characteristics questionnaire. Interviews were approximately 90 min long and were audio-recorded, then transcribed.

**Table 1:** Participant characteristics by organizational leadership level.

Participant Characteristics (*n* = 17)	Organizational leadership level, % (*n*)*		
	Mid-level	Senior-level	Total
Type of organization			
Nonprofit hospital or healthcare system	70 (7)	86 (6)	76 (13)
Community-based nonprofit organization	10 (1)	0 (0)	6 (1)
Local public health department	20 (2)	14 (1)	18 (3)
Organizational role(s)^†^			
Community or population health	60 (6)	57 (4)	76 (13)
Marketing or public relations	30 (3)	14 (1)	24 (4)
Strategic planning	30 (3)	14 (1)	24 (4)
Executive leadership	0 (0)	14 (1)	6 (1)
Education level			
College degree	30 (3)	14 (1)	24 (4)
Master’s degree	70 (7)	71 (5)	71 (12)
Doctorate degree	0 (0)	14 (1)	6 (1)
Race and ethnicity^‡^			
Black/African American (non-Hispanic)	20 (2)	0 (0)	12 (2)
White/Caucasian (non-Hispanic)	80 (8)	86 (6)	82 (14)
Hispanic/Latino	0 (0)	14 (1)	6 (1)
Gender			
Male	10 (1)	29 (2)	18 (3)
Female	90 (9)	71 (5)	82 (14)
Total	100 (10)	100 (7)	100 (17)

*Percentages may not add up to 100 due to rounding.

^†^Total organizational roles exceed *n* = 17 as participants selected one or two choices.

^‡^Race/ethnicity is self-reported.

### 2.4. Qualitative data collection and analysis

This study applied a grounded theory research approach to collect qualitative interview data, recognizing that data collection and analysis are taking place at the same time, informing one another to construct a theory that describes the phenomenon being studied (65, 66). Our analysis occurred concurrently with data collection, and this iterative process allowed us to detect questions for inclusion in pending interviews (e.g., How would repeal of the ACA influence your decision to conduct a CHNA?). Analytic notes, or memos, to describe patterns and relationships emerging in the data were also taken during these meetings. Following completion of the interviews, leadership interviews were transcribed by research assistants and subsequently reviewed by the Co-Investigator.

The data analysis team consisted of the Principal Investigator, Co-Investigator, research consultant, and one research assistant. Three members of the research team used NVivo to engage in line-by-line open coding of a sample of interviews and then met to review and synthesize codes, developing a codebook that we each used to code a subset of interviews. The larger research team met periodically throughout this process to discuss emerging codes, synthesizing codes into categories and sub-categories that described characteristics of CHNA implementation (e.g., use of vendors, involvement of the marketing department). These categories were then reviewed using a constant comparative method to describe, analyze, and evaluate possible approaches to accomplish HTCC’s stated objective of increasing the quality of primary data in Tarrant County. The research team developed a technical report with descriptive findings and evidence-based recommendations for HTCC to achieve their strategic goal. This report presented findings and recommendations to their governance board in accordance with specific questions identified by the Steering Committee (e.g., Why do organizations do a CHNA? What are the challenges in the quality of primary data?). Recommendations were driven by an implicit assumption of hospital willingness to financially contribute to collaborative data collection. Accordingly, we advised that HTCC invest in a county-focused advisory group to guide evaluation activities and advance structures for collaborative implementation. The HTCC board of directors opted not to pursue these recommendations in the subsequent rounds of federally mandated hospital reports (2018–2019).

Considering this decision, a constructivist grounded theory framework was applied as an extension of the initial analysis. Constructivist grounded theory positions qualitative findings as provisional and subject to revision, recognizing that knowledge is co-constructed in an ongoing and discursive manner (67). This framework acknowledges the co-authors’ role in shaping the interpretations, experiences, and meanings of participant interview data. We draw on an interdisciplinary practice and research lens, applying our backgrounds in public policy, public health, and social work to interpret research findings and consider implications in the context of Medicaid nonexpansion and limited CHNA collaboration. Through a re-reading of interview transcripts, memos, and descriptive categories generated in the initial coding stage, we re-immersed ourselves in the data to explore factors that inhibited CHNA collaboration among nonprofit hospitals and their partners within an urban context of Medicaid nonexpansion. With this new question in mind, the lead author drew on a grounded theory coding paradigm to regroup codes into categories and sub-categories that describe CHNA implementation decisions in terms of causal conditions, strategies, intervening conditions, and consequences (68). Emerging themes were reviewed and discussed with the research team until consensus was reached.

## 3. Results

When the decision to complete a CHNA is made, Tarrant County-based nonprofit health and public health systems carry out six distinct sets of activities: (1) define the process, (2) identify their community, (3) collect data, (4) prioritize needs, (5) determine action plans, and (6) implement community benefit activities. Key informants described CHNA implementation as an ongoing process driven by various sources of accountability in organizations with limited capacity for in-depth data collection. Participants described complex business and regulatory environments that discourage CHNA implementation decisions which fail to promote efficient resource use, administrative simplicity, align with existing service delivery structures, or respond to accountability guidelines. Outside of these factors, willingness to collaborate in CHNA implementation was contingent on tangible benefits of collaboration such as meaningful evaluation metrics for community benefit activities, data ownership, and a sense of collective impact. These themes are summarized in Table 2.

**Table 2:** Qualitative interview themes.

Theme	Description
Responding to sources of public accountability	Accountability and compliance to federal and state tax collection agencies, social and political institutions, and public trust as driving factors for completing a CHNA
Prioritizing efficiency	Strategies for CHNA implementation that minimize duplicative activities and align with existing structures and resources
Aligning with healthcare service delivery	Context and business considerations of healthcare service delivery as intervening conditions that shape implementation strategies
Establishing benefits of collaboration	Mutually beneficial consequences of CHNA collaboration as incentives for expanding partnerships

### 3.1. Responding to sources of public accountability

The scope of CHNA implementation is determined by public accountability to various stakeholders including federal and state tax collection agencies, social and political institutions, and community residents. Participants from private and public health systems differed in describing the public stakeholders that prompted the decisions to do a CHNA, as well as the breadth and scope of their roles in the data collection processes.

#### 3.1.1. Role of regulatory agencies

While both private and public hospital and health system leaders discussed their roles as stewards of public dollars, private nonprofit hospital leaders have differing fiscal responsibilities than public health departments. Public health departments are not subject to the same public regulations, and their community health assessments are associated with voluntary pursuit of national public health accreditation guidelines. Nonprofit hospital participants explained that federal guidelines required active collaboration with tax experts to honor the intent of the mandate and comply with new regulations. This was particularly challenging given delays in the finalization of IRS guidelines following the passage of the ACA. In private health systems, decision-makers acknowledged the possibility of investing resources in collaborative data collection for “boots-on-the-ground” community health data. However, collaboration appeared contingent on alignment with existing regulations.

[If the ACA is repealed] would we still do a CHNA? Yes. Would it look the same? Nope …I can definitely tell you that there are others [hospitals] around that wouldn’t at all…. If you said the state didn’t require it and the federal government didn’t require it, we would do it because we’re a steward [of tax dollars], but it wouldn’t be as driven as it is.

#### 3.1.2. Conservatism and inclusivity

The conservatism of local social and political institutions appeared to have a dampening effect on the willingness of private health systems to advance costly or controversial health initiatives. One private health system employee described their employer as a “very financially conservative, very risk-averse” healthcare organization in their approach to community health engagement by avoiding new clinical trials or programs. “We are a Christian organization so it’s making sure that you are not offending the people who are your base, your core. We live in…a very conservative state….”

In contrast, decision-makers in public health and hospital systems emphasized ideas of inclusivity in measuring and responding to community health needs. One person explained how county leaders chose to “bite off a lot” in their CHNA to be inclusive of the whole county. “We obviously aren’t touching every person, but that’s what we are responsible for.” County employees also described themselves as indirect beneficiaries of community health improvement activities.

Basically, anybody that lives, works, or plays in [this county] is a stakeholder. My job is to make sure that our stakeholders make good, healthy decisions. I work in [this county], but I don’t live here. However, I still consider myself a stakeholder of this community.

### 3.2. Prioritize efficiency

In selecting CHNA implementation strategies, decision-makers in both public and private health systems sought to comply with multiple organizational regulations and priorities. This occurred by prioritizing administrative efficiency and reducing logistical burdens of CHNA implementation through deliberate alignment with existing structures, activities, and programs.

#### 3.2.1. Limited capacity for data collection and analysis

Many participants described how past ability to collect and analyze granular CHNA data to identify community health disparities was hindered by staff inexperience and limited federal guidelines. Participants provided widely ranging definitions for concepts of population health and community health, and many noted that federal regulations provided scarce guidance in defining targeted communities, selecting data collection methods, prioritizing needs, and evaluating community benefit impact. Community input was gathered through surveys, “listening sessions,” “community councils” or “focus groups,” but persons did not generally apply the terms “qualitative” or “quantitative” to describe this data. When asked to distinguish between primary and secondary data, one participant noted, “I’ve never thought of it, that term, because I do not have a background of some statistician or epidemiologist or anything.” When implemented, health initiatives generally lacked specific outcomes or evaluation activities, and many participants noted a reluctance to engage in cross-hospital CHNA collaboration without a shared understanding of CHNA terminology and processes.

Several participants struggled with identifying and selecting survey tools and survey questions for data collection processes. Concerningly, in some cases, this limited data collection from racially and ethnically minoritized groups and persons of low socioeconomic status. “We did not get all the people, and it was obvious. Because looking at the data it was like, yeah, that’s—our neighborhoods aren’t just white, educated people…” Another participant noted the human capital toll of extended efforts to gather a more representative sample.

I think the intent and the passion is to [collect meaningful data that can drive decisions], but I think sometimes we just get overwhelmed and frustrated…I think after a while, there may be a pressure to say, you know we've been working on this…[CHNA] plan for six months.

#### 3.2.2. Administrative simplicity

In large healthcare systems, oversight of CHNA implementation occurred within corporate offices rather than individual hospital facilities. Data collection was generally overseen by persons in marketing and patient recruitment with knowledge of neighborhood demographics and ongoing involvement with community stakeholders. Community surveys and focus groups were largely drawn from convenience samples of hospital staff and community councils, professional networks, and social media. This also required collaboration across hospital departments to avoid duplication with accreditation and strategic planning effort, and hospital patients were excluded from data collection activities to avoid interference with quality improvement activities.

Most organizations relied on vendors to outsource time-consuming data collection. In one case, this included a telephone survey company with trained staff and software to collect survey data. Even with vendor support, CHNAs create burdensome demands on employees and participants, contributing to “stakeholder fatigue” from multiple forms of data collection within individual CHNAs, as well as across CHNAs for different local health systems. Extended engagement in data collection activities also appeared to diminish the quality of action plans and final CHNA reports. One health systems leader described how they reduced the workload required for CHNAs for three hospitals in the same subregion. “[The CHNAs] look exactly alike… It was kind of like, change the name.” Some participants even suggested that the desire to align with existing programs discouraged prioritization of needs that could not be addressed within existing service delivery structures.

I mean it is all made up anyway, right?… We have a [department] within our organization which has federal and state resources for services to older people, people with disabilities… and we are collaborating with [the county mental health authority], so we said we want to leverage the resources that are here.

#### 3.2.3. Practical alignment

To reduce duplicative, fragmented, or disjointed efforts, participants indicated that collaborations for higher-quality primary data would need to align with other organizational factors that defined the CHNA process, such as available resources and accountability sources. One participant described how varying sources of organizational accountability and ways of defining their local communities might hinder collaborative data collection.

We [could] all put money in to hire some entity to kind of draft the questions or do the data collection and then there is stuff like printing and marketing and some entities may have more money for that…[But] I don’t know if a community-wide assessment that has lots of partners is going to satisfy [each hospital’s CHNA requirements].

Obtaining leadership buy-in was seen as a critical aspect of advancing collaborative initiatives. One participant described integrated diverse organizational perspectives through vertical and horizontal communication. “We have an in-house group of cross-functional leaders, from all the divisions so, from top to middle level, we got their ideas as well, and they might have come up with some things that we did not like.” Another noted that investing in community benefit activities often aligned with the personal interests of organizational leaders. “A lot of our CHNA [community benefits spending] is sponsorships. A lot of those are political. Somebody sits on this board, so we have to give money to this organization.”

### 3.3. Aligning with healthcare service delivery

The CHNA process is consistently shaped by the context of healthcare service delivery and associated business priorities. While these considerations did not drive the decision to conduct a CHNA or explicitly define strategies, decision-makers noted the influence of healthcare delivery as an intervening condition that shaped key implementation decisions.

#### 3.3.1. Context of healthcare services

Rather than drawing on community health frameworks to drive CHNA implementation, participants explained how key decisions reflected alignment with the context of health care service delivery. For example, the concept of “community health” was described in terms of patient care and healthcare delivery settings, rather than distinct activities, and community health priorities were framed as disease prevalence rather than structural barriers to health. Defining communities was challenging for healthcare systems with multiple facilities in overlapping geographic boundaries, and some turned to clinical patient data to define their communities based on geographic subregions and build on existing data collection structures.

We knew we did not want to do individual CHNA’s…and [somebody] proposed…determining zones based on 75% of the inpatient and outpatient data and finding the common zip codes in those areas…they had common services lines, common patients, common geographic area.

Decision-makers also prioritized data gathered from existing clinical health services (e.g., hemoglobin counts) or service delivery activities (e.g., avoidable hospitalizations) in prioritizing needs and developing action plans for community benefit activities.

[We see] people coming to the emergency department and then getting admitted because they are not managing their diabetes. That’s a good correlation to…see here that there’s a high concentration of people with diabetes in this area… So there’s gotta be, you know, a social determinant of health, something that is not matching up to where these people are not able to manage their diabetes or choose not to.

#### 3.3.2. Business of healthcare

Investment in CHNA data collection is partly driven by local healthcare markets. As hospital facilities compete for commercially insured patients, participants described how lower profit margins drive expenses toward patient recruitment. In this environment, spending on community benefit activities is influenced by anticipated revenue from expanding patient pools. Several participants noted an interest in partnering with managed care organizations for interventions to address social determinants of health, describing this as an opportunity to cost-effectively develop community health promotion activities that could be scaled out from hospital service lines into community settings. Others preferred investing in community benefit activities with anticipated cost-savings resulting from “managing people through the community.” Consistent with definitions of “community health” as patient care in community-based settings, this included the expansion of physician health services through electronic health records, “televisits,” or community-based healthcare facilities. Participants perceived the potential of these services to provide a competitive advantage in accessing commercially insured patients while serving as a convenient vehicle for implementing community benefit activities.

We already know that people who are educated about their disease state, like pre-diabetes [have better outcomes], so if you can manage those people and manage that [condition], then you can save everybody a lot of money…It just made sense for us to put a [healthcare] facility there that could be a part of the community.

At the same time, competitive business margins may disincentivize CHNA data collection in communities with high uninsurance rates. When unrepresentative of the community, primary data provided limited guidance for identifying community benefit activities. The presence of a public hospital system may further discourage the willingness of private health systems to invest in data collection that captures local health disparities. One participant drew a sharp distinction between the responsibilities of public and private nonprofit hospitals in describing the decision to gather more targeted community health data.

In a perfect world, I could get zip-code level data…that redefine what the needs are and how they compare to each other… But we’re not [the local public hospital] … At the end of the day, we’re a business and we’re trying to get patients.

Participants from healthcare systems with a greater market share indicated greater flexibility in allocating resources for more in-depth CHNA data collection processes. One participant from a private health system described how limited competition in the local healthcare market enabled leaders to hire staff with extensive research training, develop their own surveys, apply statistical sampling methods, and develop community collaborations for disseminating data.

If we were one of 10 community hospitals in the 6-county area with each of us competing for the same patient population [of people with private health insurance], I seriously doubt our margins would be as great…and our flex spending capacity would be much smaller.

### 3.4. Benefits of collaboration

Despite the challenges associated with inclusivity, capacity, and motivation for higher-quality primary data, participants generally acknowledged the desire for higher quality primary data. When asked to consider the possibility of collaborative CHNA primary data collection, participants discussed potential “consequences” or benefits of collaboration that could influence future decisions to invest time and resources into this process, determine action plans, and evaluate program effectiveness.

#### 3.4.1. Data ownership

Some leaders emphasized the value of the CHNA process itself as a means to promote public ownership through community inclusion in planning, facilitation, and data collection. One county health leader explained, “We had to make sure that we were being very clear and transparent… that we are facilitating the [CHNA] process. This belongs to the community. We are doing this together.” By contrast, a decision-maker in a private healthcare system reflected an individual, rather than a collective, sense of ownership for community health. “Under a contract, I own [the data collected by outside vendors]. At the end of the survey, you are gonna send me the raw data…I do not want reports, I want data.”

#### 3.4.2. Measuring value

To add value in determining action plans and evaluating the impact of community benefit activities, participants suggested that collaborative primary data collection would need to move beyond merely documenting local health disparities to also assess the causes of those disparities and identify measurable targets of change. Primary data can establish useful and practical metrics for shaping organizational decisions to cap, reduce, or reallocate internal resources. One mid-level manager with experience in both public and private hospital systems noted their concerns with current and past decisions of organizational leaders to sustain certain community benefit activities in the absence of measurable community health improvement. “If it’s not meeting the mark, if you do not have a good return on your investment, leadership needs to be more stringent and say, sorry we cannot do this.” Organizational leaders frequently described the importance of maximizing both cost-efficiency and cost-effectiveness for CHNA data collection. This was consistently expressed in terms of the potential value of such data, as illustrated by the similar wording employed by two participants from distinct health care systems:

The questions [and data collected] were great, but…. the granularity wasn't there to be able to inform us, A, what to do and B, if we did anything, did it work?

If the data and the information that we are able to procure from the CHNA is of value, and we can show the value of it, then I think that it will have legs and really make an impact. If the data is not good, and it doesn’t have value, then why do it?

#### 3.4.3. Collective impact

In some cases, collaboration at the data collection stage was seen as a gateway for partnership development that could extend into community input and community benefit activities. Collaboration across health systems was viewed as a relationship-building activity that did not require “earth-shattering” conversations to support partnerships outside of CHNA implementation, such as advocacy and grant-seeking.

When we sit down as a group and collect stuff together and talk about it, it is amazing how that will continue into, 'You know what? Maybe we should try doing this together.’ It's almost human nature that you're working together.

More voices are more powerful than one…If all the hospitals…in [the county] went to certain individuals and said here’s an issue, this is why people aren’t getting healthier because they can’t get to their appointments, you have to do something about it…

## 4. Discussion

This multi-method participatory action research study revealed challenges and opportunities for greater collaboration among decision-makers responsible for CHNA implementation in an urban county in Texas, a Medicaid nonexpansion state. While participants generally upheld the value of community health and importance of addressing social determinants of health, there appears to be little interest in active collaboration for CHNA activities that fail to respond to public accountability guidelines, clearly align with existing health care services, promote administrative simplicity, or ensure efficient use of limited resources. Persons responsible for CHNA implementation are often housed within multi-county corporate health systems, placing business considerations and administrative efficiencies at the forefront of key implementation decisions. This includes, for example, decisions to contract with external vendors for the CHNA data collection process (69), and prioritizing community health needs that are congruent with existing health care services and community benefit activities of other system hospitals at the expense of more pressing community health needs (70). While some have called for supplemental system-wide CHNAs to better document and address regional needs (71), this may dilute county- or neighborhood-level needs and discourage public-private partnerships as community benefit dollars are distributed across ever-growing healthcare service regions.

Public health departments are not subject to the CHNA mandate, but many choose to engage in community health assessments as part of national accreditation guidelines (72). In contrast to their private sector counterparts, key stakeholders within public hospitals and public health departments consistently stressed the public ownership of community health assessment data and expressed a deep sense of personal and professional accountability to engage in inclusive, widespread primary community health assessments. At the same time, organizational leaders in public health systems seem to reflect limited accountability for implementing and evaluating community health efforts based on identified community health needs, emphasizing that this responsibility—much like the data collected—belonged to the public. Unlike public entities, private hospitals can retain private ownership of raw data from community health needs assessment and leverage this data for other economic activities. Additional research is needed to explore how differing regulations and sources of accountability influence willingness to establish public-private partnerships that apply needs assessment data for community health interventions. Since action plans and community benefit activities in private nonprofit hospitals may be closely aligned with political and economic priorities of hospital board members and other key stakeholders, future studies can explore how social and political interests of corporate health system boards and public health officials influence management decisions associated with CHNA processes (73).

**Figure 1:**
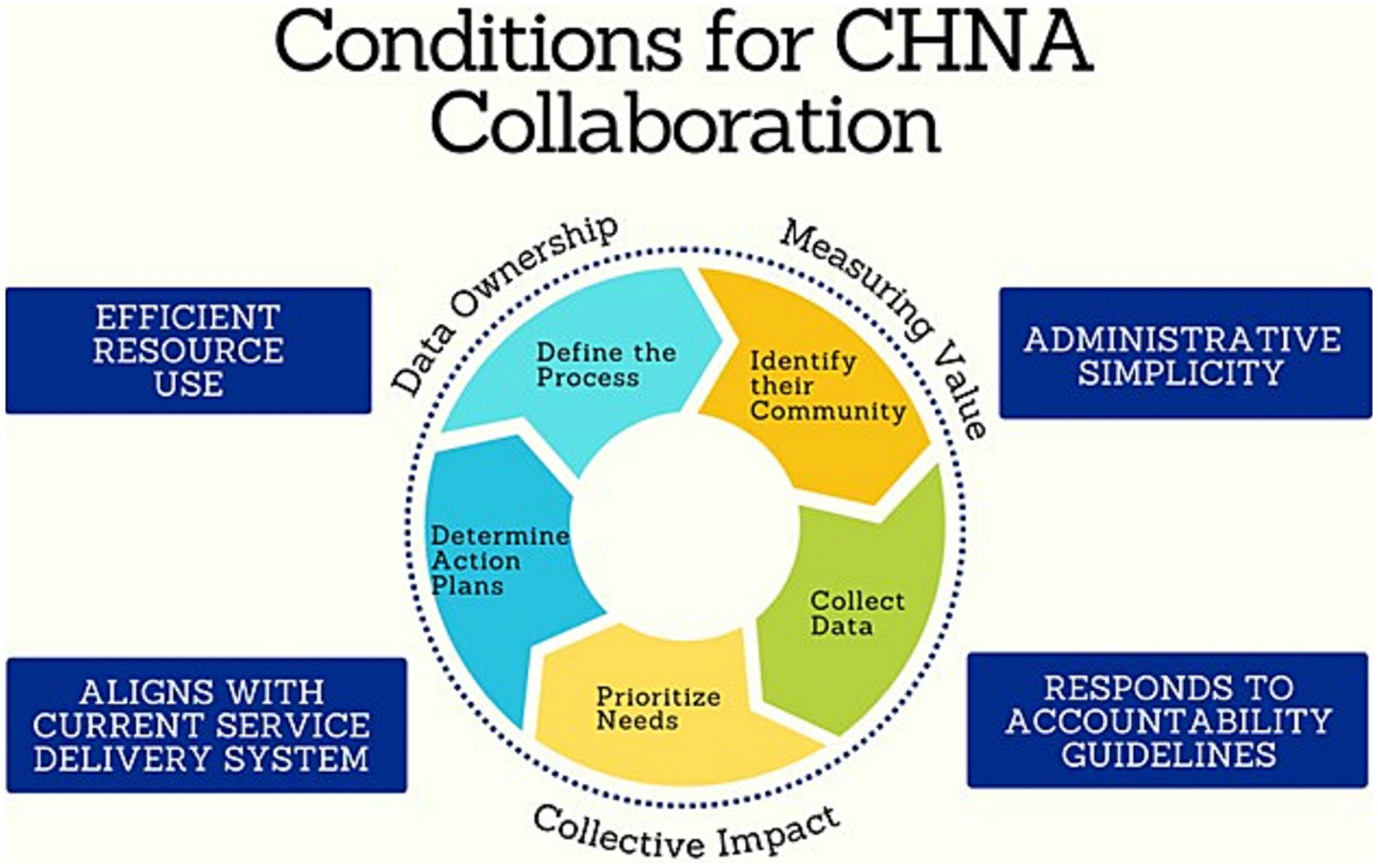
Conditions for CHNA collaboration.

To encourage greater collaborative CHNA efforts across public and private health systems, community health scholars should advance data collection strategies that reduce contextual barriers to participation and promote conditions for collaboration, as illustrated conceptually in Figure 1. This figure elaborates the grounded theory that emerged from this study, positioning CHNA collaboration as a set of mutually beneficial conditions (data ownership, measuring value, collective impact) that result from implementation strategies which respond to accountability guidelines, encourage efficient resource use, are administratively simple to implement, and align with health and social service delivery systems. For example, research and evaluation experts in anchor institutions can provide the administrative structure for efficient, rapid, and rigorous community health data collection approaches such as rapid ethnographic assessments (REAs) and environmental scans (74). REAs are a cost-efficient strategic planning tool to analyze both internal (e.g., structural and budgetary) and external (e.g., political and social) factors that impact healthcare delivery (75). This team-based applied research approach can be tailored to geographic regions of varying scope and density, allowing healthcare leaders to retain ownership of needs assessment data, assess their socio-environmental context, and document existing community engagement efforts (76). REAs can also serve as strategic windows of opportunity to nurture active engagement of public and private sector employees and build social networks for responding to an emerging health crisis (77). Translational health scholars can draw on REA findings to evaluate and recommend evidence-based community health interventions that are compatible with the organizational environment (78). This might be based on factors such as intervention components and administrative considerations, relevant social impact measures such as reductions in emergency department utilization rates (79), and budgetary or financial considerations (80).

To minimize harms associated with a competitive healthcare environment, translational health scholars can leverage hospital CHNAs to pursue structured, short-term collaborations with health systems, managed care organizations, and community-based organizations. These mutually beneficial partnerships can improve their knowledge and confidence in addressing social determinants of health (81) and build social capital for collaboration to develop and evaluate community health initiatives based on overlapping priorities (82). Health service researchers can also advance implementation and evaluation guidelines that align with electronic health record “z codes” to capture social determinants of health (83), advancing such locally tested interventions as community benefit activities that hospitals can cost-effectively expand beyond the managed care context (84).

Given the recent trend in Tarrant County politics that shifted elected leadership from moderate conservatives to more right-wing Republicans (57), these findings provide an opportunity for local scholars to continue exploring collaborative CHNA strategies in socially and politically conservative localities. Hospitals that employ health equity as a guiding theme in their CHNAs appear more likely to prioritize healthcare access and health disparities (85), but our study indicates that health care leaders in nonexpansion states may be reticent to elevate health equity frameworks perceived to misalign with economically conservative state policies, private sector engagement, or political ideology of personal responsibility (24, 38). This study suggests that scholars interested in identifying health disparities among uninsured and underinsured populations should consider promoting health equity as a less explicit by-product of the CHNA process. This might include, for example, incorporating language such as the oft-heard phrase “a hand up, not handout” to describe community health activities in the context of self-sufficiency (86). Healthcare leaders in conservative areas can also explore working with nonprofit organizations such as HTCC to facilitate coordination of needs assessments and community benefit activities. By including the local public health department as a partner, rather than facilitator, of such partnerships, this type of partnership can minimize public resistance associated with an expanded role for local government.

Previous studies have explored the use of community-based participatory research methods as a framework to conduct community-led needs assessments (41, 87, 88), but this study is unique in its application of participatory research methods within a county-based collaboration of nonprofit hospitals, public health departments, and anchor academic institutions. This study was limited to public and private nonprofit health systems in one urban Texas county and findings may not apply to CHNA implementation processes in other urban regions. While data are limited to the first two rounds of CHNA implementation following the ACA, we anticipate findings would hold true for subsequent CHNAs as they are consistent with more recent findings on barriers to higher-impact CHNAs such as unclear federal guidelines, limited staff capacity (89) and inconsistent methodologies (54).

The COVID-19 pandemic elevated the science of community engagement and sustainable partnerships as a public funding priority (90), and community health champions have a unique window of opportunity to monitor health disparities associated with the 2020–2023 public health emergency. Through the CHNA process, public health advocates can promote greater hospital accountability to identify and respond to social and health disparities associated with the COVID-19 pandemic (12, 91, 92). The persistence of state Medicaid nonexpansion and continued politicization of healthcare expansion necessitates a closer exploration of how state and local policy environments influence nonprofit hospital investment in community health initiatives. This study outlined key considerations for community health advocates to leverage the CHNA mandate for community health improvement. Despite multiple barriers and challenges in advancing collaborative needs assessments, participants in this study widely supported the notion of more rigorous, scalable, and evidence-based approaches for primary data collection to drive needs assessments that are applicable for developing and evaluating low-cost community health interventions. As health systems prepare to engage in subsequent rounds of CHNAs, health equity champions can advance mutually beneficial partnerships that align with the policy, political, and economic contexts of local health systems.

## Data availability statement

The datasets presented in this article are not readily available because the dataset is not available for public access. Requests to access the datasets should be directed to MN, marcela.nava@uta.edu.

## Ethics statement

The studies involving humans were approved by the University of Texas at Arlington Institutional Review Board. The studies were conducted in accordance with the local legislation and institutional requirements. The participants provided their written informed consent to participate in this study.

## Author contributions

All authors listed have made a substantial, direct, and intellectual contribution to the work and approved it for publication.
